# Loss calculation and thermal analysis of ultra-high speed permanent magnet motor

**DOI:** 10.1016/j.heliyon.2022.e11350

**Published:** 2022-11-04

**Authors:** Zheng Li, Pengju Wang, Libo Liu, Qianqian Xu, Shuai Che, Lucheng Zhang, Shenhui Du, Hongjie Zhang, Hexu Sun

**Affiliations:** a School of Electrical Engineering, Hebei University of Science and Technology, 26 Yuxiang Street, Shijiazhuang, 050018, China; b School of electrical engineering, Southeast University, Nanjing, Jiangsu 210018, China

**Keywords:** High speed motor, Motor loss, Temperature field, Magneto- thermal coupling

## Abstract

The ultra-high speed permanent magnet motor (UHSPM) for hydrogen fuel cell air compressor is characterized by high speed, high motor power density, small size, and high reliability. Compared to the conventional motor, the loss per unit volume is increased and therefore the calculation of the temperature field is more important than that of conventional motors. In this paper, a UHSPM with a rated speed of 90000 r/min is designed. Firstly, a finite element (FE) model of the UHSPM is established and the losses of each part of the high-speed motor are calculated, and the calculated losses are introduced into the fluid field in the form of a heat source for motor temperature analysis. The calculated losses were introduced into the fluid field in the form of a heat source and used in the motor temperature analysis. The temperature rise was then calculated for the unidirectional and bidirectional magneto-thermal coupling (MTC) respectively. The results show that the bidirectional magneto- thermal coupling (BMTC) simulation results are about 2-3 °C smaller than the experimental measured values, which can more accurately predict the motor temperature. The measurement results verify the accuracy of BMTC, and provide basic theoretical support for the subsequent cooling optimization scheme of high-speed motor.

## 1 Introduction

Limited by the severe situation of global energy shortage and climate warming, the hydrogen fuel cell system has attracted the attention of the market with the advantages of high energy conversion efficiency, no pollution, and rapid fuel supplement, and the demand is increasing (Hosseini and Wahid, 2020; Tanç et al., 2019). As the core power of the hydrogen fuel cell air compressor, the UHSPM has the following characteristics: high efficiency, high reliability, small size, and energy saving. Based on the above characteristics, oil-free centrifugal compressors powered by UHSPM have become one of the hot spots in the field of ultra-high speed air compressors (Zhao et al., 2013). UHSPM for hydrogen fuel cell air compressor usually uses SVPWM high-frequency power supply, the fundamental frequency is between 1k and 2k, there are high harmonic content, variable load conditions, high operating temperature. Therefore, the design method, loss analysis, temperature rise calculation, and motor cooling system design of UHSPM have become new challenges and problems in this field (Gerada et al., 2013; Huang et al., 2015).

At present, the world's calculation methods for motor temperature field are mainly through the analytical method and numerical calculation method. (Yeo et al., 2017) established the FE model of MTC and the equivalent thermal network model, which were verified by the temperature field distribution calculated by the FE model and the calculation results of the equivalent thermal network model. By combining with the electromagnetic field, (Cui et al., 2017) established three kinds of motor thermal models, including thermal circuit, lumped thermal network and CFD, and realized the balance between calculation time and accuracy. (Lee et al., 2019) proposed the lumped parameter thermal network method and the design method of high power density, but only studied the temperature field and electromagnetic field independently. (Popescu et al., 2013; Lu et al., 2014) input the losses obtained from the electromagnetic model directly into a collective parametric thermal model or a finite element tool to obtain the temperature distribution, but this method is not applicable to synchronous reluctance motors equipped with permanent magnets, as the flux generated by the magnets may be strongly dependent on temperature. (Guo et al., 2013) proposed an MTC analysis method for induction motor. The calculated loss is mapped to the fluid analysis based on coupled heat transfer, and then the temperature calculation is returned to the electromagnetic field for iteration. (Mo et al., 2017) calculated the loss by an optimized calculation method. The lumped parameter method and the three-dimensional FE thermal model were used to predict the temperature of each part of the motor, but only the unidirectional MTC was considered, omitting the effect on other physical fields after the change of temperature field. (Qi et al., 2019) proposed a lumped parameter thermal network model for the thermal analysis of modular spoked permanent magnet motors, using the lumped-parameter thermal network model to analyze steady-state and the transient-state temperature rises, but this model only considered the effect of losses on temperature. After using the FEM to calculate the loss, (Zhang et al., 2017a) used the flow-thermal coupling method to calculate the temperature rise distribution. (Jiang and Jahns, 2014) proposed a purely finite element-based EM-thermal coupling model and studied SPM machine tools with three different topologies, but this approach is very time-consuming to calculate the EM-thermal field coupling. (Sun et al., 2012) combined a finite element-based EM model with a finite element-based or computational fluid dynamics-based thermal model for joint simulations. The two models are not directly connected and the data exchange is manual, although the method is more accurate, it is time-consuming and inefficient. (Dong et al., 2019) proposed a coupled simulation based on iterative magneto-thermal-flow field to address motor losses, but did not consider the effect of temperature on motor steel. It takes a long time to use the CFD method to calculate the temperature distribution of the motor. (Luu et al., 2020) used the envelope parameter method to analyze and calculate the overall motor temperature rise without considering the effect on the stator material during the coupling calculation, which was not suitable for motors with serious uneven loss distribution and complex flow field. (Zhang et al., 2015a) combined electromagnetic FE analysis with a thermal resistance network to study the electromagnetic and thermal properties of the motor under different working conditions but ignored the influence of the end effect on the overall motor loss. (Jiang et al., 2018; Zhang et al., 2017b) updated the material properties according to the temperature distribution and verified the accuracy of the MTC analysis model. Based on the motor simulation model in electromagnetic and temperature fields, (Shi et al., 2020) carried out the magnetic-thermal coupling simulation analysis and used the bidirectional MTC method to analyze the thermal of the motor, which ensured the high calculation accuracy.

**Figure 1:**
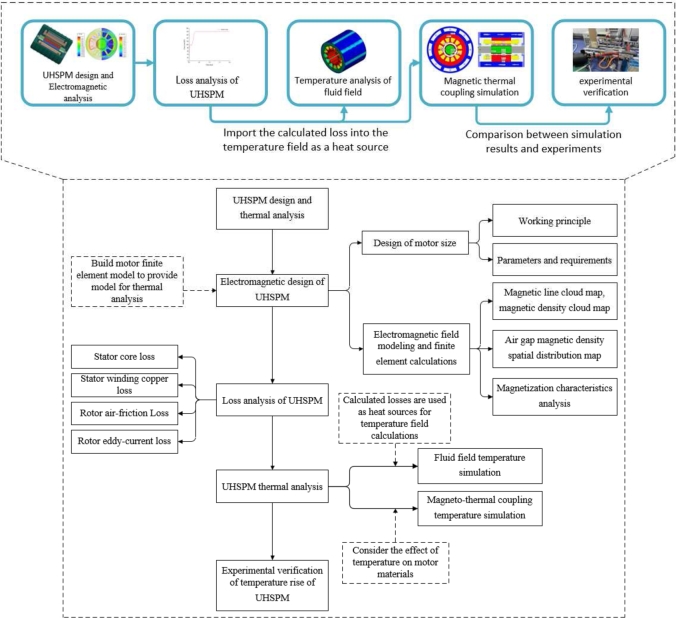
Working flowchart of whole process.

In summary, although the research on UHSPM has reached a certain level at this stage, the characteristics of the UHSPM itself tend to cause large losses and high-temperature problems, UHSPM in the loss calculation and multi-physics field MTC field is still extremely complex. Fig. 1 is the working flowchart of whole process. In this paper, a UHSPM with a rated speed of 22 kW and 90000 r/min is used as an example. And the temperature distribution of UHSPM is analyzed based on MTC, based on which the temperature rise experiments are conducted for different positions of the motor. Combining the fluid field results with the MTC simulation results and comparing the two with the experimental data, a more accurate and realistic bidirectional MTC simulation analysis of the temperature rise can be obtained, which provides the basic theoretical support for the subsequent cooling optimization scheme of UHSPM.

## 2 Electromagnetic design of motors

When the UHSPM is running, the high-frequency alternating magnetic field generates the loss of the stator core in the stator, and the introduction of the three-phase high-frequency alternating current into the stator winding also generates a large copper loss. Therefore, the focus of the stator design of the UHSPM is the selection of the stator slot type, winding, and material. Based on meeting the design parameters of the motor, the loss of the stator is minimized to facilitate heat dissipation.

**Figure 2:**
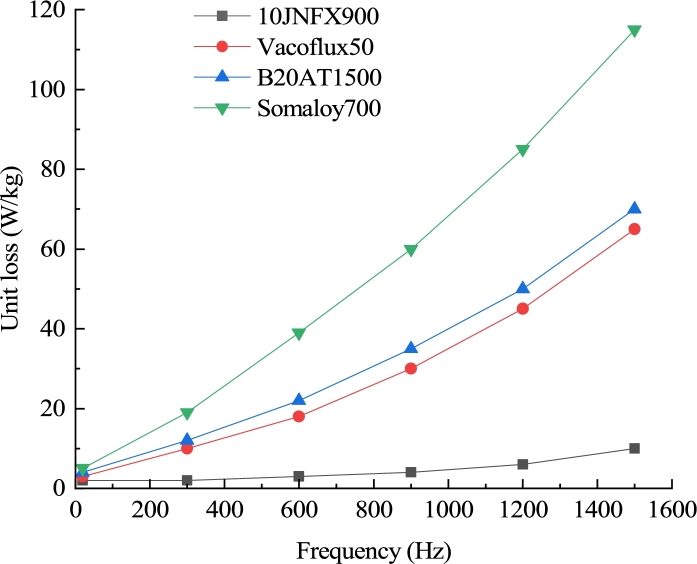
Loss-frequency curve of stator material.

When the frequency of the alternating magnetic field of the UHSPM core increases, the stator iron loss increases exponentially with the frequency by 1.5 times. When selecting stator material for UHSPM over 1000 Hz, it is necessary to select a suitable material to reduce core loss and temperature rise. Fig. 2 shows the loss-frequency curves of different stator materials of common UHSPMs. According to the comparison of curves, it can be found that the unit loss of 10JNFX900 magnet steel used in the motor in this paper is less at the same frequency, and the saturated magnetic induction intensity is as high as 1.88 T.

**Figure 3:**
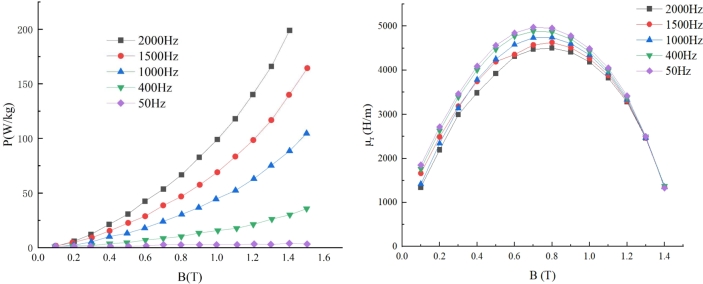
Measurement results of magnetic properties of 10JNFX900 with 0.1 mm thickness. (a) B-P curves at multiple frequencies. (b) Magnetic conductivity at multiple frequencies *μ*_r_.

Using an Epstein square circle test equipment, the magnetic characteristics of 0.1 mm thick 10JNFX900 were measured in the paper. Fig. 3 depicts the results.

From the experimental results, it can be obtained that with the gradual increase of frequency, the unit iron loss of the steel sheet increases, and with increasing frequency, the permeability steadily decreases. It can be seen that the permeability of the steel sheet under high-frequency operating conditions changes significantly, which needs to be considered in the UHSPM design process.

It is difficult to analyze the stress concentration point of the rotor of a UHSPM under super high rotation. The strength of a permanent magnet (PM) increases greatly when it is a solid structure, which is beneficial to UHSPM with a small volume. Radial magnetization produces a large iron loss, parallel magnetization is easier to get a sinusoidal distribution of air gap magnetic field, so this paper chooses parallel magnetization. As a common rotor protection measure for UHSPMs, rotor sheath uses high-strength materials to fix and protect PMs. The excellent performance of carbon fiber materials will also cause the heat loss of the rotors, which increases the risk of demagnetization of PMs at high temperatures. Therefore, titanium alloy is selected as the protective sleeve of the PM in this paper. The technical indexes of the 22 kW UHSPM to be designed are shown in Table 1.

**Table 1:** Technical indexes of 22 kW UHSPM machine.

Parameter	Value
Power rating	22 kW
Rated voltage	258 V
Number of phases	3
Rated speed	90000 r/min
Power factor	≥0.95
Efficiency	≥95%
Cooling conditions	Air cooling + coolant

In the dimension design, comprehensive consideration and multiple iterations are needed. The rated power, rated speed and volume parameters of a UHSPM need to meet the following formula (1) (Xu et al., 2019):

(1) $$D_{i}^{2}l_{ef}=\frac{6.1K_{E}P_{N}}{n_{c}\alpha_{p}\eta_{N}K_{Nm}K_{dp}AB_{a}\cos\varphi_{N}}$$

where $D_{i}$ is the inner diameter of the stator, $l_{ef}$ is the effective core length, $K_{Nm}$is the waveform coefficient of the air gap magnetic field, $P_{N}$ is the rated power, $K_{dp}$ is the winding factor, $n_{c}$ is the calculated speed, $\alpha_{p}$ is the pole arc factor, *A* is the line load, $B_{a}$ is the air gap magnetic density and $\cos\varphi_{N}$ is the power factor.

The expression of rotor centrifugal force under rated speed is (Girard et al., 2019):

(2) $$\delta =\frac{F}{S}=\rho r^{2}\omega^{2}$$

The strength of the material needs to be calculated at the peak state of the force value. After multiplying by the safety factor, the expression of the minimum strength of the material is as follows (Yamazaki and Kato, 2013):

(3) $$\delta \leq \frac{\left[\delta \right]}{C}$$

where *ρ* is the density of the rotor material, *S* is the area of the rotor cross-section, *ω* is the angular velocity of the rotor, *r* is the rotor radius, [*δ*] is the maximum material stress and *C* is the safety factor.

The rotor material parameters and the maximum rotational speed are known, the maximum value of the outermost linear speed of the rotor is obtained from the above equation and the outermost diameter of the rotor is expressed as (Liu et al., 2016):

(4) $$D_{\max}=\frac{2}{\omega }\sqrt{\frac{\left[\delta \right]}{C\rho }}$$

The main design parameters of the motor are calculated according to formula (1)–(4). The design parameters are shown in Table 2.

**Table 2:** Design parameters of 22 kW UHSPM machine.

Parameter	Value
Number of poles/slots	2/12
Stator outer diameter	104 mm
Stator inner diameter	45.3 mm
Air gap	4.5 mm
Rotor outer diameter	36.3 mm
PM outer diameter	30.3 mm
Stator core length (including end plates)	86 mm

**Figure 4:**
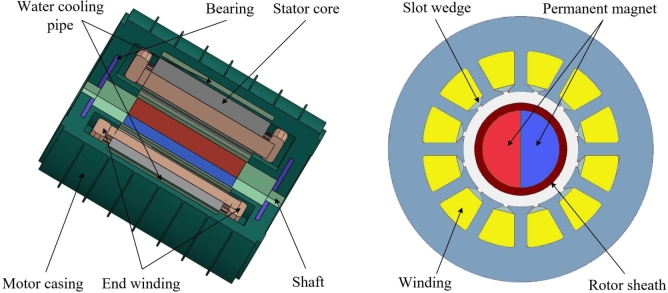
Overall structure diagram of motor. (a) Axial distribution. (b) Radial distribution.

To enhance the UHSPM's motor performance, the rotor PM adopts a Sm_2_Co_7_ PM and adopts a fixed parallel magnetization method. Fig. 4 shows the overall structure of the solid rotor UHSPM. The solid rotor has excellent strength performance, higher air-gap flux density, and higher overall efficiency than the traditional surface-mounted PM motor. This motor adopts 0.45 mm diameter ring winding, which not only reduces the length of the motor but also improves the cooling efficiency of air cooling.

**Figure 5:**
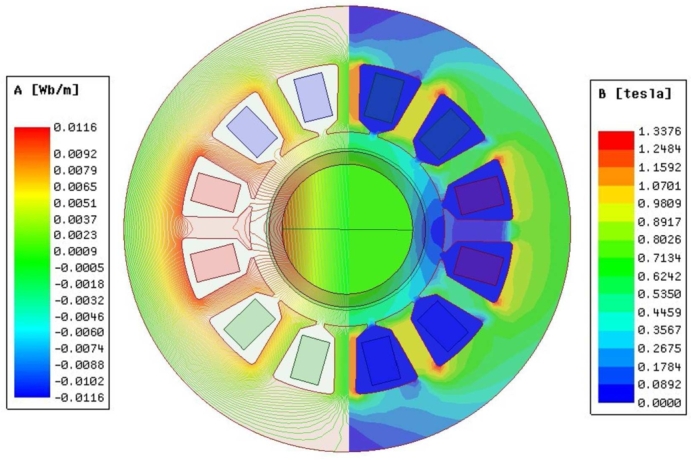
Magnetic line cloud and magnetic density cloud of UHSPM.

**Figure 6:**
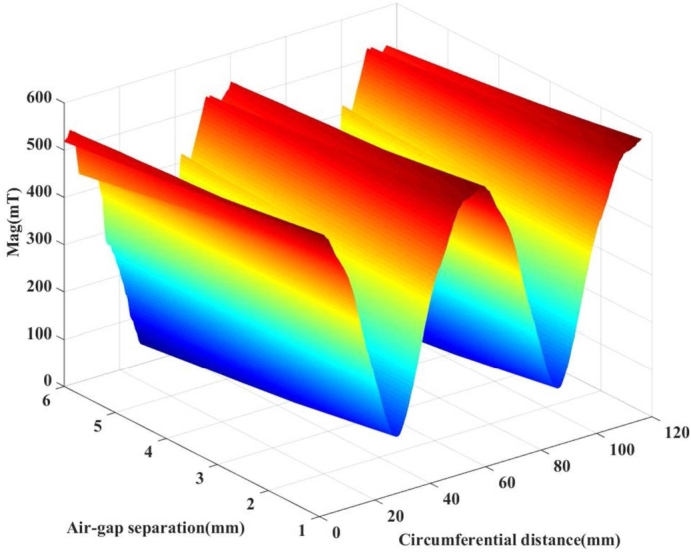
Spatial distribution of air gap magnetic density of UHSPM.

The electromagnetic property of the motor was calculated using FE software. Fig. 5 shows the magnetic line distribution cloud and magnetic density cloud of the UHSPM. The analysis of the magnetic lines shows that the stator teeth have low magnetic leakage and the coil position is biased towards the stator slot, which greatly reduces the eddy current effect and the skinning effect of the wire. The right half of the magnetic density cloud shows no saturation at the stator core, as the magnetic induction intensity of the PMs is at its maximum value. The stator teeth have a larger magnetic density distribution than the yoke, with a peak of 1.3376 T. The stator core magnetic density is lower than the magnetic saturation value of 1.8 T for silicon steel sheet material, which verifies that the stator material will not exceed saturation and meets the design requirements. Fig. 6 shows the spatial distribution of the air gap magnetic density of the UHSPM, which is observed to be sinusoidally distributed.

## 3 Calculation of motor losses

### 3.1 Stator iron losses

The stator material will produce loss under the action of the high-frequency changing magnetic field. The classical Bertotti iron loss separation calculation model divides the stator core loss $P_{\mathrm{iron}}$ into hysteresis loss $P_{h}$, classical eddy current loss $P_{c}$ and additional eddy current loss $P_{e}$, as shown in formula (5) (Okamoto et al., 2016):

(5) $$P_{\mathrm{iron}}=P_{h}+P_{c}+P_{e}$$

(6) $$P_{h}=K_{h}fB_{m}^{\alpha }$$

(7) $$P_{c}=K_{c}{\left(fB_{m}\right)}^{2}$$

(8) $$P_{e}=K_{e}{\left(fB_{m}\right)}^{1.5}$$

where $K_{h}$, *α* is the hysteresis loss factor, *f* is the alternating frequency of the magnetic field, $B_{m}$ is the magnetic density amplitude (magnetic induction intensity amplitude), $K_{c}$ is the classical eddy current loss factor and $K_{e}$ is the additional loss factor.

For the conventional PM motor, it can be seen that there is an only alternating magnetic field in the motor, and only alternating core loss is produced. However, during operation, the magnetization of the alternating magnetic field in the core causes rotational losses in the core. The rotating magnetization phenomenon cannot be ignored in the UHSPM. The analytical method decomposes the elliptical rotating magnetic field by orthogonal decomposition. The alternating magnetic field can be decomposed into the magnetic density in both long-axis and short-axis directions by resolution, which is equivalent to the complex magnetic field of the stator by synthesizing two alternating magnetic fields in an equivalent way, so the core loss of the UHSPM can be expressed by the following equation (Zhang et al., 2020):

(9) $$\begin{array}{c} P_{\mathrm{Iron}}=P_{h}+P_{c}+P_{e}=K_{h}f\left(B_{\max}^{\alpha }+B_{\min}^{\alpha }\right)+K_{c}f^{2}\left(B_{\max}^{2}+B_{\min}^{2}\right)\\ +K_{e}\frac{1}{T}\int_{0}^{T}{\left({\left|\frac{dB_{r}(t)}{dt}\right|}^{2}+{\left|\frac{dB_{\theta }(t)}{dt}\right|}^{2}\right)}^{\frac{3}{4}}dt \end{array}$$

where $B_{\max}$ is the long-axis magnetic density of the elliptical field, $B_{\min}$ is the short-axis magnetic density of the elliptical field, $B_{r}(t)$ is the radial component of the magnetic density at the point of analysis, $B_{\theta }(t)$ is the tangential component of the magnetic density at the point of analysis, and *T* is the rotational period of the fundamental field.

**Figure 7:**
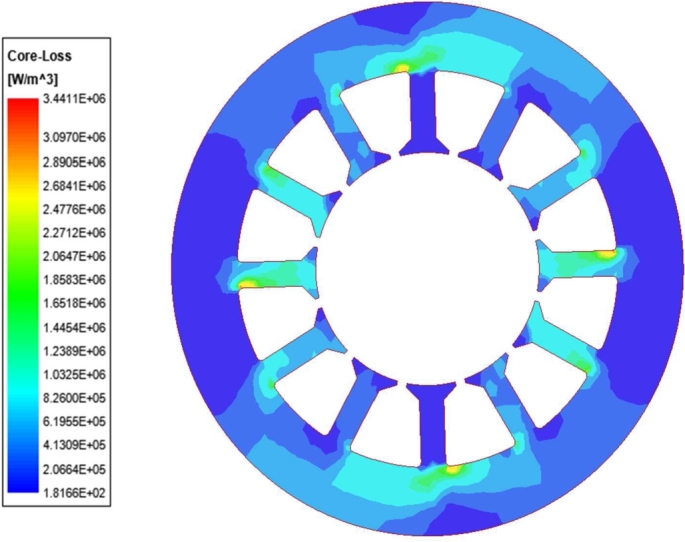
Load iron consumption cloud.

**Figure 8:**
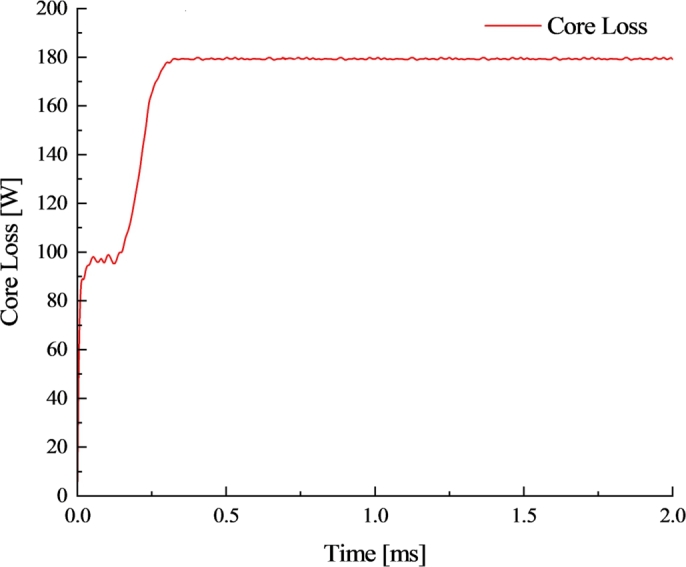
Variation curve of load iron consumption.

Bring the obtained parameters into formula (9) for analytical calculation of stator iron loss, and conduct Maxwell finite element simulation of iron loss. The stator losses are primarily concentrated in the stator teeth, as shown in Fig. 7, which is the same as the region where the maximum magnetic density is obtained in Fig. 5, this is because of the high harmonic content of the magnetic density in this region, which leads to an excessive loss density. The average value of the stator iron consumption of the motor load is 180.46 W, as shown in Fig. 8.

### 3.2 Stator copper loss

In the case of low winding current frequency, the skin effect will not have an excessive impact on the motor and can be disregarded in the calculation of losses; however, high-speed permanent magnet motors have extremely high frequencies and the frequency of the high harmonic components of the current in the winding is higher, and under the influence of the skin effect, the AC winding losses will increase large and often cannot be ignored. The skin effect can be expressed by the skin depth *δ* as follows (Malloy et al., 2014):

(10) $$\delta =\sqrt{\frac{2}{\omega_{a}\sigma \mu }}$$

where $\omega_{a}$ is the angular frequency, *σ* denotes the material's conductivity, and *μ* is the material's permeability.

In the design process of high-speed permanent magnet motor, when selecting the wire diameter, the wire diameter should be less than the penetration depth of skin effect. The rated frequency of UHSPM developed in this research is 1500 Hz and the switching frequency of frequency converter is 6000-7000 Hz, skin and proximity losses are in-creased due to high frequency current ripples induced by PWM. But the wire diameter in this paper is 0.45 mm, which is less than the penetration depth of skin effect, which can greatly reduce the additional eddy current loss of stator winding caused by skin effect. The copper consumption of stator winding of high-speed motor can be expressed as (Huynh et al., 2009):

(11) $$P_{\mathrm{AC}}=P_{\mathrm{DC}}+P_{\mathrm{add}}$$

where $P_{\mathrm{AC}}$ is the AC loss, $P_{\mathrm{DC}}$ is the DC loss and The increased eddy current loss is denoted by $P_{\mathrm{add}}$.

The formula for calculating DC losses $P_{\mathrm{DC}}$ is shown in formula (12) (Chen, 2000):

(12) $$P_{\mathrm{DC}}=mI^{2}R$$

The additional eddy current losses are shown in formula (13) (Gao and Yu, 2013):

(13) $$P_{\mathrm{add}}=P_{\mathrm{DC}}\left(k_{d}-1\right)$$

where,

(14) $$k_{d}=\varphi (\xi )+\left[\frac{N^{2}-1}{3}-{\left(\frac{N}{2}\sin\left(\frac{\gamma }{2}\right)\right)}^{2}\right]\psi (\xi )$$

(15) $$\varphi (\xi )=\xi \frac{\sinh(2\xi )+\sin(2\xi )}{\cosh(2\xi )-\cos(2\xi )}$$

(16) $$\psi (\xi )=2\xi \frac{\sinh(\xi )-\sin(\xi )}{\cosh(\xi )+\cos(\xi )}$$

where $k_{d}$ is the average resistance coefficient, *γ* is the phase angle of the upper and lower windings in the double-layer winding, *ξ* denotes the relative height of the conductors degree, and *N* is the total number of conductors in the double-layer winding.

According to the analytical calculation of formulas (11)–(16), the copper consumption of the motor stator is 240.35 W.

### 3.3 Rotor surface wind wear losses

The rotor shape used in this paper is cylindrical, and the rotor's air friction loss can be computed using the formula (17)–(20) (Fang et al., 2018):

(17) $$P_{f}=kC_{f}\pi \rho \omega^{3}r^{4}L$$

where *k* is the surface roughness coefficient of the rotor, the coefficient of friction is $C_{f}$, *ρ* is the air density, *ω*, *L*, *r* are the rotational angular velocity, axial length and cylinder radius.

The expression for the coefficient of friction $C_{f}$ on the surface of a rotating cylinder is (Huang et al., 2015):

(18) Cf=0.0153Reδ[1+(32Rea7Reδ)2]0.38

The Reδ and Rea in Equation (18) are calculated as follows:

(19) Reδ=ρωrδμ

(20) Rea=ρva2δμ

where Reδ is the radial Reynolds number, representing the loss degree caused by the friction between the rotating body and the air; Rea is the axial Reynolds number, representing the loss degree caused by the friction between the cooling fluid and the outer surface air of the rotating body; *δ* is the air gap size, *μ* is the air viscosity, $v_{a}$ is the axial forced air cooling wind speed. Through the classical wind friction loss calculation formula (17), the wind friction loss value of the rotor is 480.44 W.

### 3.4 Rotor Eddy current losses

Due to the skin effect, eddy current losses are concentrated on the outer surface of the sheath, and this part can represent the overall high frequency losses of the rotor PM. This research employs Maxwell to simulate the eddy current loss of the motor since the accuracy of the analytical approach for estimating the eddy current loss of the PM has to be improved. The magnetic field equation can be expressed as (Han et al., 2020):

(21) $$\nabla \times \frac{1}{\mu_{r}}(\nabla \times A_{m})=J_{s}-\sigma_{c}\left(\frac{\partial A_{m}}{\partial t}+\nabla V\right)+\nabla H_{c}$$

where $A_{m}$ is the magnetic vector potential, $J_{s}$ is the source current density, *V* is the potential scalar, $\mu_{r}$ is the relative permeability, $\sigma_{c}$ is the conductivity of the material, and $H_{c}$ is the coercivity of the permanent magnet.

According to Ampere's law, the total current density can be expressed by (Niu et al., 2012):

(22) $$J_{t}=-\sigma \left(\frac{dA}{dt}-\nabla V\right)$$

In the solution of two-dimensional electromagnetic field, the vector magnetic potential has only the z-axis component. i.e. $A_{x}=A_{y}=0$. The PM's eddy current loss density is calculated using formula (23) (Zhang et al., 2017a):

(23) $$P_{E}=L\iint_{S}\frac{{\left|J_{Z}\right|}^{2}}{\sigma }dS$$

where $J_{Z}$ is the z-direction component of current density.

Maxwell Finite Element Calculation by formula (21)–(23), the eddy current loss of the motor sheath and PM is 37.6 W, the motor's overall loss is 938.55 W, the rated power is 22 kW, and the motor efficiency is >95%, which meets the design requirements.

## 4 UHSPM temperature field calculations

### 4.1 Establishment of the temperature rise model and setting of boundary conditions

The mathematical model of temperature field is shown in formula (24)–(27). Without taking into account the change in temperature increase coefficient as a function of location, the differential equation of heat conduction can be expressed as formula (24) (Du et al., 2019):

(24) $$\rho c_{p}\frac{\partial T}{\partial t}=\lambda_{bx}\frac{\partial^{2}T}{\partial x^{2}}+\lambda_{by}\frac{\partial^{2}T}{\partial y^{2}}+\lambda_{bz}\frac{\partial^{2}T}{\partial z^{2}}+q_{v}$$

where $\lambda_{bx}$, $\lambda_{by}$ and $\lambda_{bz}$ are the thermal conductivity in the *x*, *y* and *z* directions respectively, $q_{v}$ is the density of the heat source, *ρ* is the density of the material and the material's specific heat capacity is denoted by $c_{p}$.

The three types of boundary conditions commonly used are (Tong et al., 2021):

(25) $${T|}_{S_{1}}=T_{c}(x,y,z)$$

(26) $$-{\lambda_{b}\frac{\partial T}{\partial n}|}_{S_{2}}=q_{0}(x,y,z)$$

(27) $$-{\lambda_{b}\frac{\partial T}{\partial n}|}_{S_{2}}=\alpha_{3}\left(T-T_{f_{3}}\right)$$

where $T_{c}(x,y,z)$ is the temperature on boundary $S_{1}$, $q_{0}(x,y,z)$ is the heat flux on boundary $S_{2}$, $\alpha_{3}$ is the heat dissipation coefficient on boundary $S_{3}$, and $T_{f_{3}}$ is the fluid temperature around $S_{3}$.

Considering the complexity of the thermal simulation analysis of the UHSPM, to ensure the correctness of the simulation calculation results, the following essential assumptions are made:

1) The effect of thermal radiation from individual components is not considered;

2) Only the steady state heat dissipation is calculated under the rated operating conditions of the motor.

**Figure 9:**
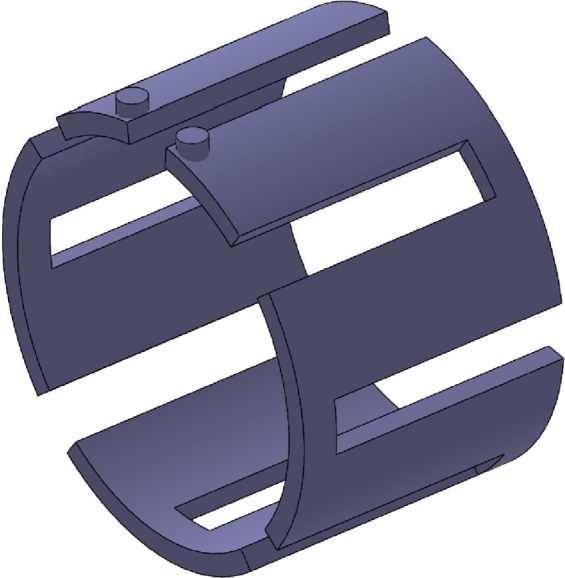
The schematic diagram of waterway structure.

In this paper, the stator of the UHSPM is axially water-cooled, the model is shown in Fig. 9, and the rotor is air-cooled with the stator inner diameter. Assuming that the air-rotor contact is an adiabatic wall, the convective heat transfer coefficient between the housing of the UHSPM and the environment is selected to be 22 W/m^2^K (Baojun et al., 2022). The motor boundary conditions are set as shown in Table 3.

**Table 3:** Motor boundary conditions.

Cold in-water temperature	Cooling water flow rate	Air cooling flow	Air-cooled inlet air temperature	Ambient temperature
65 °C	6 L/min	240 g/min	80 °C	45 °C

### 4.2 Fluid field temperature simulation

**Figure 10:**
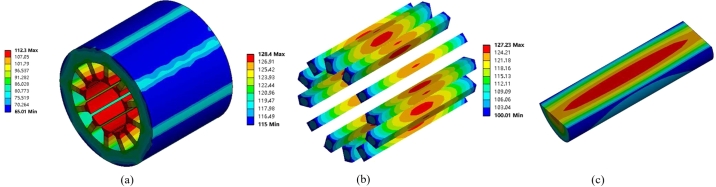
Motor temperature distribution. (a) Stator temperature. (b) Winding temperature. (c) PM temperature.

The loss obtained from the above calculation is employed as the heat source for each section of the motor. Fig. 10 depicts the outcomes of the calculations.

The highest temperature of the stator part in Fig. 10(a) is 112.3 °C, in which the stator temperature increases gradually from outside to inside, which is because the motor's housing is water-cooled, and the heat dissipation outside the stator is better, while the internal part of the motor adopts air-cooling, but the heat dissipation is relatively poor, and the heat generation of the winding and rotor parts inside the motor will be dissipated from inside to outside. The temperature at the two ends of the winding is lower in Fig. 10(b) than in the center, because the two ends of the winding are in direct contact with the air, while the middle part of the winding is in contact with the stator core, and the heat needs to be transferred from the stator core, while the heat at the two ends of the winding is directly taken away by the air, and the temperature is lower compared with the middle part of the winding. The greatest temperature region of the solid rotor PM is near the center, as shown in Fig. 10(c), which is 127.2 °C, and the outer surface has a lower temperature due to the ventilation relationship.

## 5 MTC temperature simulation analysis

### 5.1 MTC analysis method

**Figure 11:**
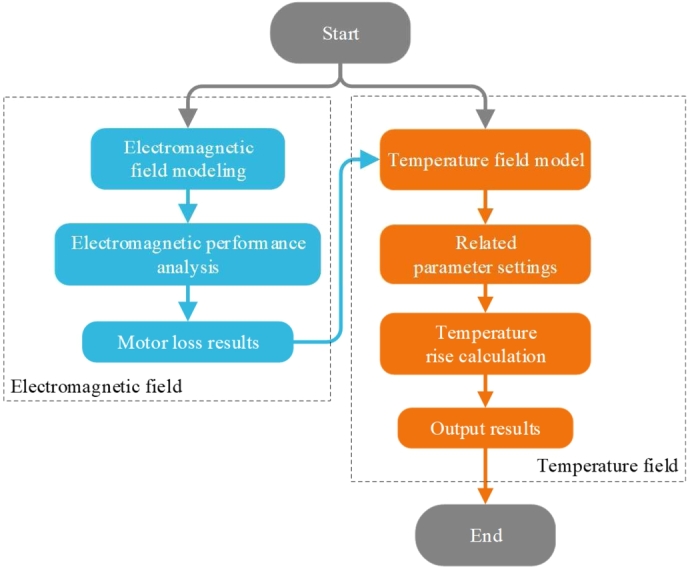
Unidirectional MTC analysis flow chart.

**Figure 12:**
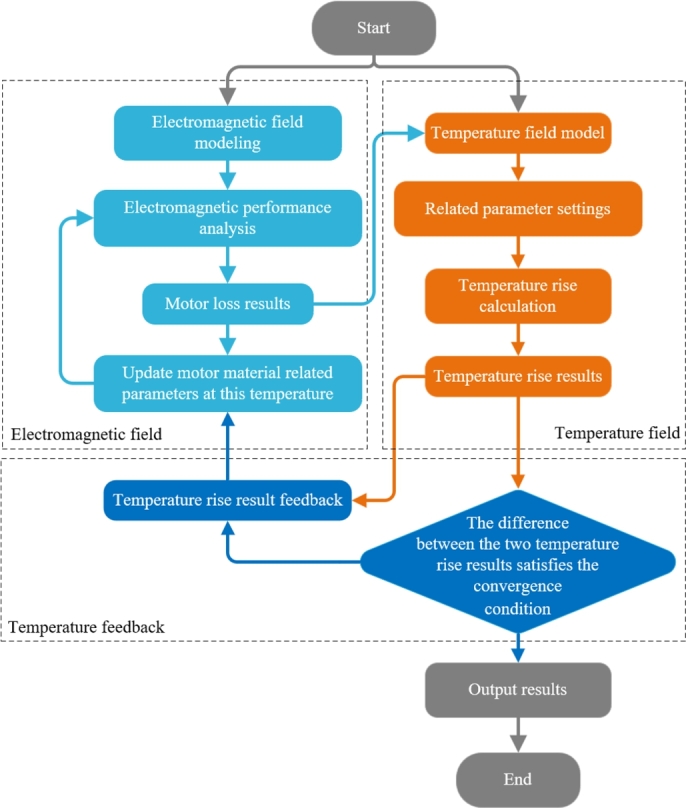
Bidirectional MTC analysis flow chart.

The calculation method of fluid field temperature rise usually defines the material properties in the simulation calculation as a fixed value, ignoring the influence of temperature on material properties, which is prone to large errors and large deviations between simulation results and experimental values. In this section, the Unidirectional MTC calculation is carried out first. Compared with the fluid field temperature calculation in the previous section, the unidirectional MTC can reduce the occurrence of inaccurate temperature calculations caused by the uneven artificial distribution of heat sources. The specific calculation flow is shown in Fig. 11. Then the Bidirectional MTC calculation is carried out, as shown in Fig. 12, and the properties of the motor material are defined as the amount that varies with the temperature, with specific effects as shown in Formula (28)–(30). The amount of change of material properties in the temperature field simulation calculation is provided to the electromagnetic calculation in real-time, which makes the temperature rise calculation more accurate after several iterations (Zhang et al., 2015b).

### 5.2 Influencing factors of MTC temperature calculation

(1) The effect of temperature on the resistivity of PMs

The eddy current effect of the PM material in UHSPM generates an important factor of heat, and the size of its resistivity affects the eddy current loss to some extent. The corresponding relation equation is shown in the following equation (28) (Ruoho et al., 2010):

(28) $$\rho_{m}=cT+d$$

where $\rho_{m}$ is the resistivity of the PM, *c*, *d* are material-related coefficients, and *T* is the temperature.

(2) The effect of temperature on the magnetism of PMs

The magnetic field in the UHSPM is mainly a combination of the armature reaction and the magnetic field fixed by the PMs. Therefore, a change in the state of the PMs will certainly affect the overall magnetic field of the UHSPM, and the corresponding losses will change accordingly. Since the magnitude of iron consumption is related to the state of the stator magnetic field, and the variation of iron consumption caused by the change of temperature characteristics of the PMs will affect the temperature calculation of the motor. The relationship between PM characteristics and temperature is shown in equation (29) below (Zhou et al., 2012):

(29) $$\left\{ \begin{array}{c} B_{{\mathrm{rt}}_{1}}=\left(1-\frac{\mathrm{IL}}{100}\right)\left[1+\left(t_{1}-t_{0}\right)\frac{\alpha_{\mathrm{Br}}}{100}\right]B_{{\mathrm{rt}}_{0}}\\ H_{{\mathrm{ct}}_{1}}=\left(1-\frac{\mathrm{IL}}{100}\right)\left[1+\left(t_{1}-t_{0}\right)\frac{\alpha_{\mathrm{Hc}}}{100}\right]H_{{\mathrm{ct}}_{0}} \end{array}\right.$$

where *IL* is the irreversible loss rate of $B_{r}$, $B_{{\mathrm{rt}}_{0}}$ is the remanent magnetization at an ambient temperature of $t_{0}$ °C and $H_{{\mathrm{ct}}_{0}}$ is the calculated coercivity at an ambient temperature of $t_{0}$ °C. $\alpha_{\mathrm{Br}}$ and $\alpha_{\mathrm{Hc}}$ are the inverse temperature coefficients of $B_{{\mathrm{rt}}_{0}}$ and $H_{{\mathrm{ct}}_{0}}$, and $t_{1}$ is the Running temperature.

(3) The effect of temperature on winding coils

The material of the motor stator winding is metallic copper, and the copper consumption of the motor winding is a component of the motor temperature increase, and the magnitude of the corresponding loss value is very important for the influence of the UHSPM temperature field variation. The proportional relationship between the coil resistance value and the motor temperature is shown in the following equation (30) (Si et al., 2018):

(30) $$\frac{R_{t2}}{R_{t1}}=\frac{234.5+T_{t2}}{234.5+T_{t1}}$$

where $R_{t1}$ and $R_{t2}$ are the winding resistance values at $T_{t1}$ and $T_{t2}$ temperatures respectively.

### 5.3 MTC temperature calculation

**Figure 13:**
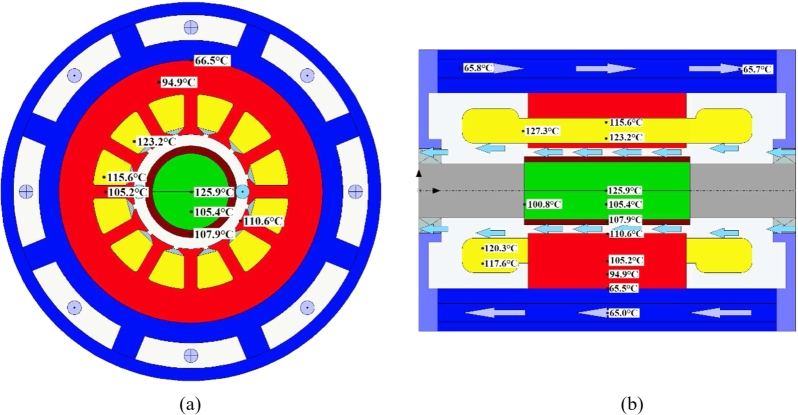
Unidirectional MTC temperature results.

The temperature distribution obtained from the unidirectional coupling temperature rise calculation is shown in Fig. 13, wherein, Fig. 13 (a) is the radial temperature diagram and Fig. 13 (b) is the axial temperature diagram.

Compared with the above three-dimensional FE fluid field temperature calculation results, the temperature obtained from the Unidirectional MTC temperature calculation is somewhat lower, but the overall temperature distribution trend of the obtained motor is the same, i.e., the rotor PM temperature is relatively high, and the motor temperature gradually decreases from the inside to the outside. The peak temperature of the PM region is 125.9 °C and the peak temperature of the stator is 110.6 °C, as calculated by the unidirectional MTC temperature.

**Figure 14:**
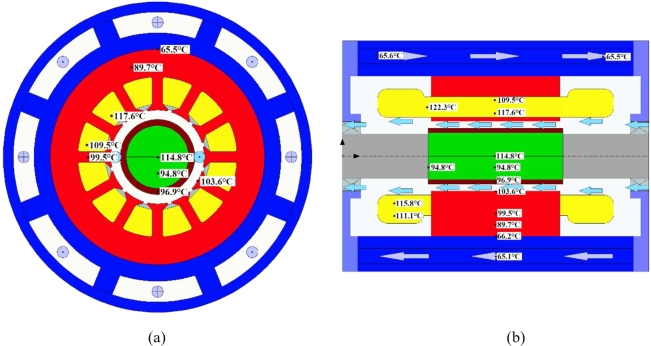
Bidirectional MTC temperature results.

Fig. 14 shows the temperature calculation results of bidirectional coupling, wherein, Fig. 14 (a) and Fig. 14 (b) are the radial and axial temperature diagram. The temperature of the key nodes differs from the unidirectional calculation results by a maximum of 9.66%, which meets the error requirement of 10%, and the temperature value of each part is slightly reduced, and the temperature rise trends calculated by comparing the fluid fields are approximately the same. The peak temperature in the PM region in the bidirectional MTC temperature calculation is 114.8 °C, and the maximum temperature of the stator is 103.6 °C. From the comparison graphs of unidirectional and bidirectional MTC temperatures, it can be seen that the calculated losses of the two methods differ due to the temperature calculation influence factors considered for bidirectional coupling, so the corresponding temperature changes will also deviate. When the motor reaches steady-state operation, the temperature state of the important parts of the UHSPM is in a reasonable range and meets the motor design specifications.

### 5.4 Experimental analysis

**Figure 15:**
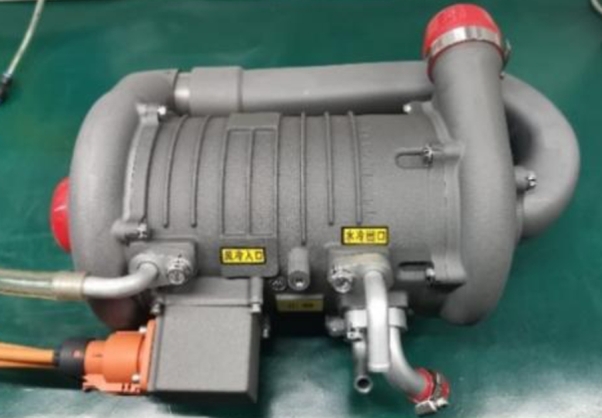
22 kW hydrogen fuel cell air compressor prototype.

**Figure 16:**
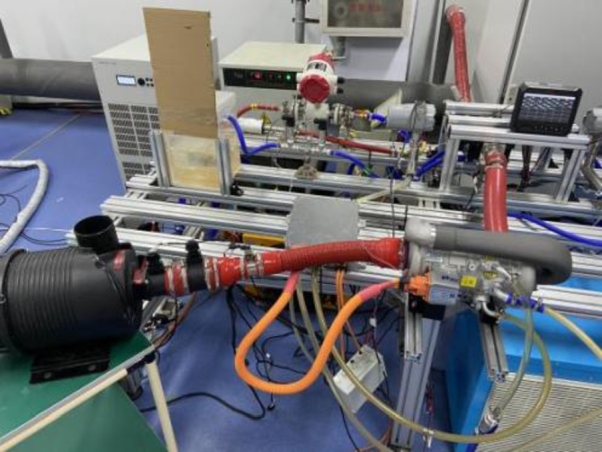
Test platform.

Fig. 15 and Fig. 16 show the UHSPM experimental prototype and the built test platform, respectively.

The stator temperature was monitored in real time using the XMTD-7332P temperature controller and a pt100 thermistor pre-built in the motor winding. Because of the extremely high speed of the UHSPM, there are some problems in measuring the rotor temperature of the motor, so the rotor temperature rise is not considered in this paper.

**Figure 17:**
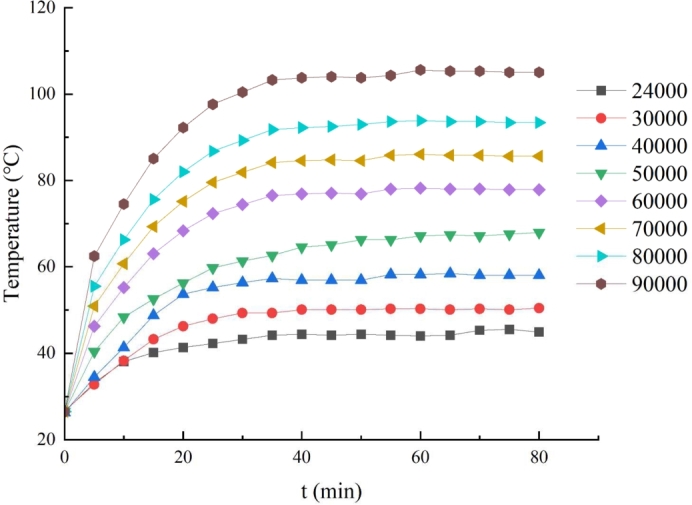
Variation curve of stator temperature at different speed.

**Figure 18:**
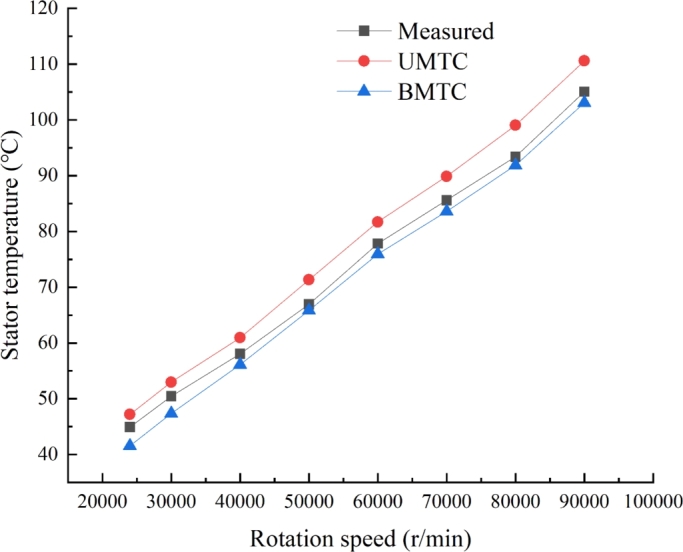
Comparison of the stator temperature rise calculated and measured for the motor at different speeds.

The UHSPM was tested at 24000, 30000, 40000, 50000, 60000, 70000, 80000 and 90000 r/min. The temperature of the thermistor pre-installed in the motor winding was recorded every 5 minutes from the start of the motor operation for each speed. The variation of the stator temperature with time at different speeds is given in Fig. 17, which shows that the stator temperature reaches a steady state after 70 minutes. When the speed is 90,000 r/min, the stator measured temperature is about 105 °C. Fig. 18 shows the experimentally measured UHSPM stator temperature variation at different speed states. From the measurement results, the measured value of the stator temperature coincides most steadily with the results of the Bidirectional MTC analysis, with the calculated value being about 2-3 °C smaller than the measured value. Because the calculated result of core loss is smaller than the actual result is an important reason, and in the actual motor there are stray losses; in addition, there is a certain current in the actual no-load operation of the motor, but the current is considered to be zero in the simulation calculation.

## 6 Conclusion and future research

In this paper, a 22 kW, 90,000 r/min UHSPM was designed for the calculation of the MTC temperature rise of the UHSPM. The work and results of this paper are discussed below.

(1) The main loss calculation is performed. The rotor surface wind wear loss accounts for the largest proportion, followed by stator copper loss and stator iron loss, and rotor eddy current loss is the least.

(2) The temperature field calculation model of HUSPM is established, and the temperature rise of fluid field and magneto thermal coupling is calculated. The calculation results show that the temperature distribution trend is the same. The difference between the bidirectional coupling simulation results and the experimental data is only 2-3 °C, which verifies the accuracy and effectiveness of the temperature rise calculation method.

In summary, this paper provides the basis and direction for temperature prediction and cooling mode optimization through accurate calculation of motor temperature rise distribution. For future research, more in-depth loss calculation of the motor can get more accurate motor temperature (Zhuo et al., 2021). We will conduct more in-depth research on motor loss methods to ensure that the motor temperature calculation model is more accurate.

**Table:** 

List of symbols and description	
Symbol	Description

*A*	Line load
*A*_m_	Magnetic vector potential
*B*_a_	Air gap magnetic density
*B*_max_	Long-axis magnetic density of the elliptical field
*B*_min_	Short-axis magnetic density of the elliptical field
*B*_r_(*t*)	Radial component of the magnetic density at the point of analysis
*B*_*θ*_(*t*)	Tangential component of the magnetic density at the point of analysis
$B_{{\mathrm{rt}}_{0}}$	Remanent magnetization at an ambient temperature of *t*_0_ °C
*B*_*m*_	Magnetic density amplitude
*C*	Safety factor
*C*_*f*_	Coefficient of friction
*c*	Material-related coefficients
*c*_p_	Material's specific heat capacity
*D*_i_	Inner diameter of the stator
*f*	Alternating frequency of the magnetic field
*H*_*c*_	Coercivity of the permanent magnet
$H_{{\mathrm{ct}}_{0}}$	Calculated coercivity at an ambient temperature of *t*_0_ °C
*IL*	Irreversible loss rate of *B*_r_
*J*_*s*_	Source current density
*J*_Z_	Z-direction component of current density
*k*	Surface roughness coefficient of the rotor
*K*_h_	Hysteresis loss factor
*K*_*Nm*_	Waveform coefficient of the air gap magnetic field
*K*_*dp*_	Winding factor
*K*_c_	Classical eddy current loss factor
*K*_e_	Additional loss factor
*k*_*d*_	Average resistance coefficient
*l*_*ef*_	Effective core length
*L*	Axial length
*N*	Total number of conductors in the double-layer winding
*n*_c_	Calculated speed
*P*_*N*_	Rated power
*P*_iron_	Stator core loss
*P*_h_	Hysteresis loss
*P*_c_	Classical eddy current loss
*P*_e_	Additional eddy current loss
*P*_AC_	AC loss
*P*_DC_	DC loss
*P*_add_	Increased eddy current loss
*q*_0_(*x*,*y*,*z*)	Heat flux on boundary *S*_2_
*q*_v_	Density of the heat source
*R*_t1_	Winding resistance values at *T*_t1_ temperatures respectively
*R*_t2_	Winding resistance values at *T*_t2_ temperatures respectively
*r*	Rotor radius
*r*_c_	Cylinder radius
Re_*δ*_	Radial Reynolds number
Re_*a*_	Axial Reynolds number
*S*	Area of the rotor cross-section
*T*	Rotational period of the fundamental field
*t*_1_	Running temperature
*T*_*t*_	Temperature
*T*_*c*_(*x*,*y*,*z*)	Temperature on boundary *S*_1_
$T_{f_{3}}$	Fluid temperature around *S*_3_
*V*	Potential scalar
*v*_*a*_	Axial forced air cooling wind speed
*α*_Br_	Inverse temperature coefficients of $B_{{\mathrm{rt}}_{0}}$
*α*_Hc_	Inverse temperature coefficients of $H_{{\mathrm{ct}}_{0}}$
*α*_*p*_	Pole arc factor
*α*_3_	Heat dissipation coefficient on boundary *S*_3_
*δ*	Skin depth
*δ*_a_	Air gap size
[*δ*]	Maximum material stress
*γ*	Phase angle of the upper and lower windings in the double-layer winding
*ρ*_d_	Density of the material
*ρ*_m_	Resistivity of the PM
*λ*_b*x*_	Thermal conductivity in the *x* directions respectively
*λ*_b*y*_	Thermal conductivity in the *y* directions respectively
*λ*_b*z*_	Thermal conductivity in the *z* directions respectively
*μ*	Material's permeability
*μ*_a_	Air viscosity
*μ*_r_	Relative permeability
*ρ*	Density of the rotor material
*ρ*_a_	Air density
*ρ*_d_	Density of the material
*σ*_c_	Conductivity of the material
*σ*	Material's conductivity
*ω*_*a*_	Angular frequency
*ω*	Rotational angular velocity
*ξ*	Relative height of the conductors degree
$\cos\varphi_{N}$	Power factor

## Declarations

### Author contribution statement

Zheng Li, Hexu Sun: Conceived and designed the experiments; Performed the experiments; Analyzed and interpreted the data; Contributed reagents, materials, analysis tools or data; Wrote the paper. Pengju Wang, Libo Liu: Conceived and designed the experiments; Performed the experiments; Analyzed and interpreted the data; Wrote the paper. Qianqian Xu, Shuai Che, Lucheng Zhang: Analyzed and interpreted the data. Shenhui Du, Hongjie Zhang: Contributed reagents, materials, analysis tools or data.

### Data availability statement

Data will be made available on request.

### Declaration of interests statement

The authors declare no conflict of interest.

### Additional information

No additional information is available for this paper.

## References

[br0010] Baojun G., Jiong Z., Tao D. (2022). Temperature prediction and cooling structure optimization of explosion-proof high pressure water-cooled double speed motor. Energy Rep..

[br0020] Cui S., Sun P., Kuang Z. (2017). IEEE Transportation Electrification Conference & Expo, Asia-Pacific (ITEC Asia-Pacific).

[br0030] Chen S.K. (2000).

[br0040] Dong B., Wang K., Han B. (2019). Thermal analysis and experimental validation of a 30 kW 60000 r/min high-speed permanent magnet motor with magnetic bearings. IEEE Access.

[br0050] Du G., Xu W., Zhu J. (2019). Power loss and thermal analysis for high-power high-speed permanent magnet machines. IEEE Trans. Ind. Inform..

[br0060] Fang H., Li D., Qu R. (2018). Rotor design and Eddy-current loss suppression for high-speed machines with a solid-PM rotor. IEEE Trans. Ind. Appl..

[br0070] Girard N., Olmedo L.E., Schiffmann J. (2019). 2019 19th International Symposium on Electromagnetic Fields in Mechatronics, Electrical and Electronic Engineering (ISEF).

[br0080] Gao N., Yu L. (2013). 2013 IEEE International Conference on Mechatronics and Automation.

[br0090] Gerada D., Mebarki A., Brown N.L. (2013). High-speed electrical machines: technologies, trends, and developments. IEEE Trans. Ind. Inform..

[br0100] Guo H., Lv Z., Wu Z. (2013). Multi-physics design of a novel turbine permanent magnet generator used for downhole high-pressure high-temperature environment. IET Electr. Power Appl..

[br0110] Han B.C., Peng S., He Z. (2020). Eddy current loss calculation and thermal analysis of high-speed motor winding in magnetically suspended control moment gyroscope. Opt. Precis. Eng..

[br0120] Hosseini S.E., Wahid M.A. (2020). Hydrogen from solar energy, a clean energy carrier from a sustainable source of energy. Int. J. Energy Res..

[br0130] Huang Z., Fang J., Liu X. (2015). Loss calculation and thermal analysis of rotors supported by active magnetic bearings for high-speed permanent-magnet electrical machines. IEEE Trans. Ind. Inform..

[br0140] Huynh C., Zheng L., Acharya D. (2009). Losses in high speed permanent magnet machines used in microturbine applications. J. Eng. Gas Turbines Power.

[br0150] Jiang Y., Wang D., Chen J. (2018). Electromagnetic-thermal-fluidic analysis of permanent magnet synchronous machine by bidirectional method. IEEE Trans. Magn..

[br0160] Jiang W., Jahns T.M. (2014). Coupled electromagnetic–thermal analysis of electric machines including transient operation based on finite-element techniques. IEEE Trans. Ind. Appl..

[br0170] Luu P.T., Lee J.Y., Lee J.H. (2020). Electromagnetic and thermal analysis of permanent-magnet synchronous motors for cooperative robot applications. IEEE Trans. Magn..

[br0180] Lee J.G., Yeo H.K., Jung H.K. (2019). Electromagnetic and thermal analysis and design of a novel-structured surface-mounted permanent magnet motor with high-power-density. IET Electr. Power Appl..

[br0190] Liu X., Hao C., Jing Z. (2016). Research on the performances and parameters of interior pmsm used for electric vehicles. IEEE Trans. Ind. Inform..

[br0200] Lu Q., Zhang X., Chen Y. (2014). Modeling and investigation of thermal characteristics of a water-cooled permanent-magnet linear motor. IEEE Trans. Ind. Appl..

[br0210] Mo L., Zhu X., Zhang T. (2017). Temperature rise calculation of a flux-switching permanent-magnet double-rotor machine using electromagnetic-thermal coupling analysis. IEEE Trans. Magn..

[br0220] Malloy A.C., Martinez-Botas R.F., Lampérth M. (2014). Measurement of magnet losses in a surface mounted permanent magnet synchronous machine. IEEE Trans. Energy Convers..

[br0230] Niu S., Ho S.L., Fu W.N. (2012). Eddy current reduction in high-speed machines and Eddy current loss analysis with multislice time-stepping finite-element method. IEEE Trans. Magn..

[br0240] Okamoto S., Denis N., Kato Y. (2016). Core loss reduction of an interior permanent-magnet synchronous motor using amorphous stator core. IEEE Trans. Ind. Appl..

[br0250] Popescu M., Dorrell D.G., Alberti L. (2013). Thermal analysis of duplex three-phase induction motor under fault operating conditions. IEEE Trans. Ind. Appl..

[br0260] Qi J., Hua W., Zhang H. (2019). Thermal analysis of modular-spoke-type permanent-magnet machines based on thermal network and FEA method. IEEE Trans. Magn..

[br0270] Ruoho S., Haavisto M., Takala E. (2010). Temperature dependence of resistivity of sintered rare-Earth permanent-magnet materials. IEEE Trans. Magn..

[br0280] Shi Y., Wang J., Wang B. (2020). Electromagnetic-thermal coupled simulation under various fault conditions of a triple redundant 9-phase PMASynRM. IEEE Trans. Ind. Appl..

[br0290] Sun X., Cheng M., Zhu S. (2012). Coupled electromagnetic-thermal-mechanical analysis for accurate prediction of dual-mechanical-port machine performance. IEEE Trans. Ind. Appl..

[br0300] Si J., Zhao S., Feng H. (2018). Analysis of temperature field for a surface-mounted and interior permanent magnet synchronous motor adopting magnetic-thermal coupling method. CES Trans. Electr. Mach. Syst..

[br0310] Tong W., Sun R., Li S. (2021). Loss and thermal analysis for high-speed amorphous metal PMSMs using 3-D electromagnetic-thermal bi-directional coupling. IEEE Trans. Energy Convers..

[br0320] Tanç B., Arat H.T., Baltacıoğlu E. (2019). Overview of the next quarter century vision of hydrogen fuel cell electric vehicles. Int. J. Hydrog. Energy.

[br0330] Xu H., Geng H., Lin H. (2019). 2019 IEEE International Conference on Mechatronics and Automation (ICMA).

[br0340] Yeo H.K., Park H.J., Seo J.M. (2017). Electromagnetic and thermal analysis of a surface-mounted permanent-magnet motor with overhang structure. IEEE Trans. Magn..

[br0350] Yamazaki K., Kato Y. (2013). Optimization of high-speed motors considering centrifugal force and core loss using combination of stress and electromagnetic field analyses. IEEE Trans. Magn..

[br0360] Zhuo L., Sun L., Shi D., Sun R., Zou J. (2021). Semi-analytical model and experimental verification of rotor Eddy current loss of high temperature high speed permanent magnet machine considering temperature change. Chin. Soc. Elecr. Eng..

[br0370] Zhang C., Chen L., Wang X. (2020). Loss calculation and thermal analysis for high-speed permanent magnet synchronous machines. IEEE Access.

[br0380] Zhang Y., McLoone S., Cao W. (2017). Power loss and thermal analysis of a MW high-speed permanent magnet synchronous machine. IEEE Trans. Energy Convers..

[br0390] Zhang G., Hua W., Cheng M. (2017). Coupled magnetic-thermal fields analysis of water cooling flux-switching permanent magnet motors by an axially segmented model. IEEE Trans. Magn..

[br0400] Zhang F., Du G., Wang T. (2015). Electromagnetic design and loss calculations of a 1.12-MW high-speed permanent-magnet motor for compressor applications. IEEE Trans. Energy Convers..

[br0410] Zhang B., Qu R., Wang J. (2015). Electromagnetic–thermal coupling analysis of permanent magnet synchronous machines for electric vehicle applications based on improved (μ+1) evolution strategy. IEEE Trans. Magn..

[br0420] Zhao D., Blunier B., Gao F. (2013). Control of an ultrahigh-speed centrifugal compressor for the air management of fuel cell systems. IEEE Trans. Sustain. Energy.

[br0430] Zhou P., Lin D., Xiao Y. (2012). Temperature-dependent demagnetization model of permanent magnets for finite element analysis. IEEE Trans. Magn..

